# Age-associated B cells in viral infection

**DOI:** 10.1371/journal.ppat.1010297

**Published:** 2022-03-17

**Authors:** Isobel C. Mouat, Marc S. Horwitz

**Affiliations:** 1 Centre for Inflammation Research, University of Edinburgh, Edinburgh, United Kingdom; 2 Department of Microbiology and Immunology, University of British Columbia, Vancouver, British Columbia, Canada; Yale University School of Medicine, UNITED STATES

## Abstract

Age-associated B cells (ABCs) are a recently identified, unique B cell population that displays both protective and pathogenic characteristics, depending on the context. A major role of ABCs is to protect from viral infection. ABCs expand during an array of viral infections and display various functional capacities, including secretion of antibodies and activation of T cells. Following resolution of infection, ABCs appear to persist and play a crucial role in memory and recall responses. Here, we review the currently understanding of ABCs in the antiviral response in both humans and mice. We discuss avenues for future research, including the impact of sex on the ABC population and heterogeneity of ABCs between contexts.

## Introduction

A unique B cell population, termed age-associated B cells (ABCs), was identified in 2011 in the contexts of aging and autoimmunity [1,2]. The frequency of ABCs increases with age, particularly in females, and is elevated during various autoimmune and autoinflammatory diseases [3]. ABCs express the transcription factor T-bet, which has been well characterized in various infections [4]. Recently, B cells broadly expressing T-bet have received much attention in potentiating antiviral immune responses, and, accordingly, it has since been shown that ABCs do in fact respond to an array of viral infections (Table 1). ABCs, atypical memory B cells, and T-bet^+^ B cells are all names used to describe what is likely a similar population, with primary markers used to denote the population including high expression of both CD11c and T-bet and low expression of CD21. The precise contribution(s) of ABCs to health and disease continues to be examined.

**Table 1:** ABCs are expanded in an array of viral infections and vaccinations. The relative proportion of ABCs is increased following various viral infections as measured by the cell markers listed and vaccinations in both mice and humans and are found at multiple anatomical locations.

**Primary viral infection**	**Virus**	**Host species**	**Cell markers (reference)**	**Anatomical location**
	HCV	Human	CD19^+^T-bet^+^ [5]	Peripheral blood
	Rhinovirus	Human	CD19^+^CD20^+^CXCR5^−^T-bet^+^ [6]	Peripheral blood, nasal tissue
	HIV	Human	CD19^+^CD27^+^T-bet^+^ [7] CD19^+^T-bet^+^ [8]	Peripheral blood, lymph nodes
	SARS-CoV-2	Human	CD19^+^CD27^−^CD38^−^CD24^−^IgD^−^CD11c^+^CD21^−^[9]	Peripheral blood
	Influenza	Mouse and human	CD19^+^IgD^−^T-bet^hi^ [10] B220^+^T-bet^+^ [11]	Spleen, mediastinal lymph nodes, lung, blood
	LCMV	Mouse	CD19^+^T-bet^+^ [12] CD19^+^CD11c^+^CD11b^+^T-bet^+^ [13]	Spleen
	Vaccinia	Mouse	CD19^+^CD11c^+^CD11b^+^T-bet^+^ [13]	Spleen
	MCMV	Mouse	CD19^+^CD11c^+^CD11b^+^T-bet^+^ [13]	Spleen
	γHV68	Mouse	CD19^+^CD11c^+^CD11b^+^T-bet^+^ [13]	Spleen
**Vaccination**	Influenza	Human	CD19^+^CD38^lo^CD27^+^CD21^lo^ [14] CD19^+^T-bet^hi^CD21^lo^CD27^−^[15]	Peripheral blood
	Yellow fever	Human	CD19^+^CD27^+^T-bet^+^ [7]	Peripheral blood
	Vaccinia	Human	CD19^+^CD27^+^T-bet^+^ [7]	Peripheral blood

ABC, age-associated B cell; HCV, hepatitis C virus; HIV, human immunodeficiency virus; LCMV, lymphocytic choriomeningitis virus; MCMV, murine cytomegalovirus; SARS-CoV-2, severe acute respiratory syndrome coronavirus 2; γHV68, gammaherpesvirus-68.

The frequency of ABCs has been shown to increase in various viral infections in humans and mice, including hepatitis C virus (HCV), rhinovirus, human immunodeficiency virus (HIV), severe acute respiratory syndrome coronavirus 2 (SARS-CoV-2), influenza, lymphocytic choriomeningitis virus (LCMV), vaccinia, murine cytomegalovirus (MCMV), and gammaherpesvirus-68 (γHV68) [5–13] (Table 1). The proportion of circulating ABCs is also increased following several vaccinations in people, including those for influenza [14,15], yellow fever [7], and vaccinia [7] (Table 1).

ABCs are a major population of virus-specific B cells during infection and following vaccination [6–8,10,14,16]. In individuals with HIV, nearly all of the B cells specific to HIV envelope glycoprotein gp140 in the peripheral blood are ABCs [7]. Following influenza infection in mice, the majority of virus-specific B cells in the lungs are ABCs 15 days postinfection, and one-third of virus-specific B cells in the spleen are ABCs 100 days postinfection [10]. Following influenza vaccination, a significantly higher proportion of ABCs are specific for the influenza surface protein hemagglutinin compared to classical memory B cells [14]. Evidence suggests that ABCs are long-lived effector B cells that display memory characteristics and are poised to differentiate into an antibody-secreting cell upon challenge [14].

ABCs display distinct activation requirements, transcriptional profile, and localization patterns compared to other B cell subsets [1,2,10]. Their enrichment for antigen specificity as well as persistence and effector functional capacities indicate that ABCs likely play an important role during and following viral infections (Box 1). There is much to be learned about this unique population during health, infection, and disease. Here, we briefly review what is currently known about the role of ABCs in viral infections and discuss possibilities for future investigations.

**Box 1. Learning points**

- Age-associated B cells (ABCs) increase during viral infection and following vaccination.
- Following infection, ABCs remain elevated long term and are important for robust recall responses.
- ABCs secrete antiviral antibodies and cytokines and stimulate T cells.
- How antiviral ABCs relate to ABCs during autoimmune disease and aging remains incompletely understood.

### Anatomical distribution

**Fig 1:**
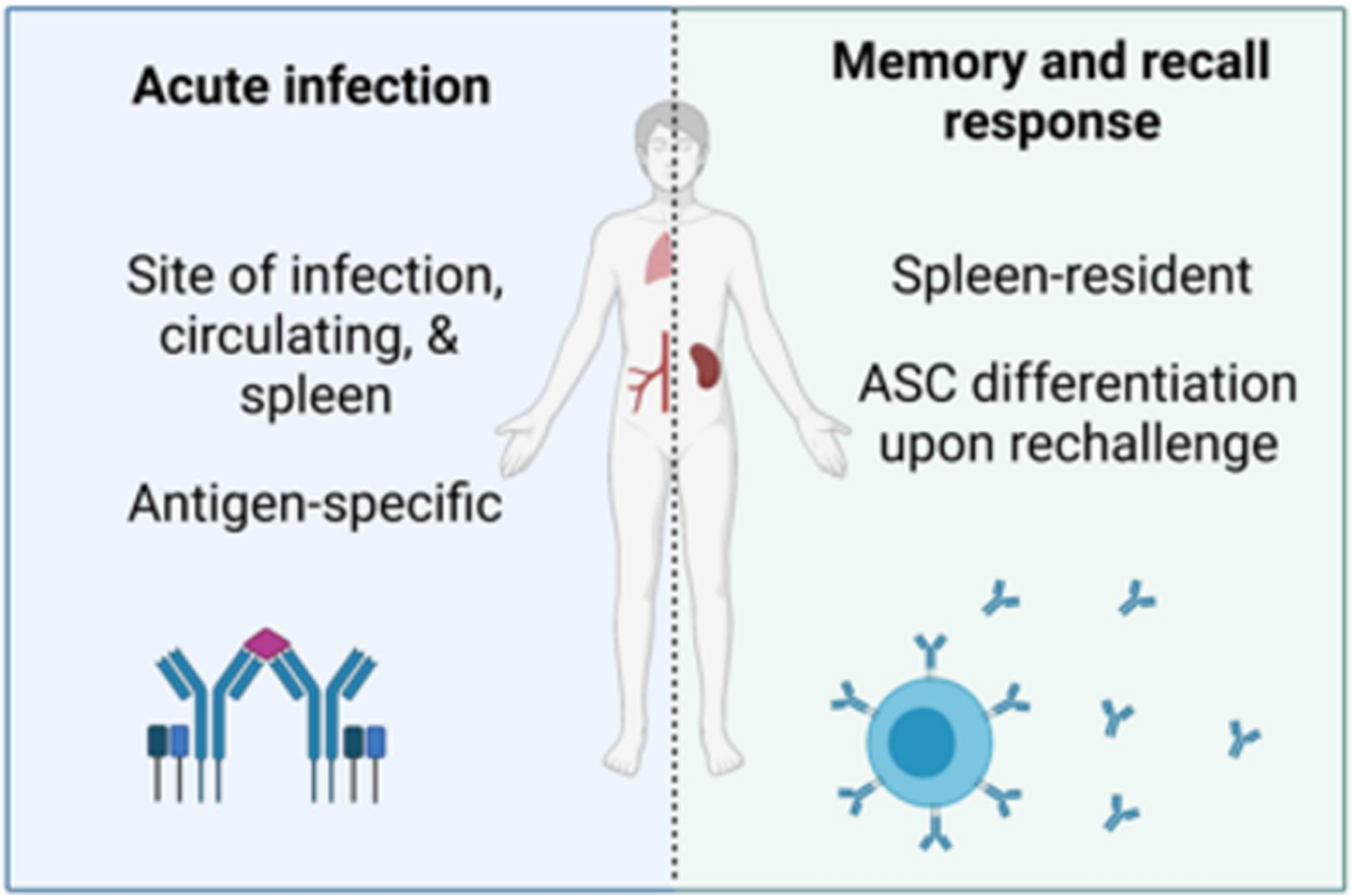
ABCs in acute infection and recall response. During acute viral infection, ABCs are increased at the site of infection, in circulation, and in the spleen, and are largely antigen specific. Following clearance of acute infection, ABCs primarily reside in the spleen and differentiate into antibody-secreting cells upon rechallenge. The figure was created using BioRender.com. ABC, age-associated B cell; ASC, antibody-secreting cell.

During acute or active viral infection, ABCs expand both in the spleen and at the site of infection (Fig 1 and Table 1). In both humans and mice, influenza infection results in increased ABC frequency in the lungs and the mediastinal lymph nodes (LNs), in addition to the spleen [10]. In individuals with HIV viremia, ABCs expand in LNs [8], and during rhinovirus infection, ABCs are elevated in the blood and nasal tissue [6]. The frequency of ABCs then decreases at the site of infection following its resolution. After clearance of acute influenza infection in mice, the number and proportions of ABCs is decreased in the lung, mediastinal LNs, and blood, as compared to active infection [10]. In individuals with HCV, the frequency of circulating ABCs is significantly decreased following antiviral treatment and clearance of the infection, compared to the frequency at the onset of treatment [5].

Current evidence suggests that ABCs are predominantly maintained in the spleen following resolution of infection (Fig 1). ABCs remain in the spleen at an elevated frequency long-term (100+ days) in mice infected with influenza compared to naïve mice [10]. Mouse parabiosis experiments demonstrate that antigen-specific ABCs are spleen resident and do not circulate systemically during steady state conditions [10]. The anatomical distribution of these ABCs primarily residing in the spleen during homeostasis appears largely conserved between mice and humans; in healthy individuals, ABCs are typically found in the spleen and bone marrow, though also at low numbers in tonsils and LNs [10]. Collectively, these studies indicate that the frequency of ABCs is increased in the spleen and at the site of disease during ongoing infection and that ABCs persist primarily in the spleen following clearance.

### Differentiation

The signals that stimulate ABC differentiation are induced via the synergistic engagement of various cytokine and antigenic receptors that are activated during viral infection, including the interferon gamma (IFNγ) receptor, Toll-like receptor 7 (TLR7), and the B cell receptor [13,17–22]. It has not been conclusively determined from which B cell populations ABCs arise. While follicular B cells have been shown to be able to differentiate into ABC-like cells under appropriate conditions ex vivo [23], other subsets, including marginal zone or transitional B cells, could also contribute to the ABC pool in vivo. ABCs display diversity in germ line V_H_ and Vκ genes, indicating they are not a single clonally expanded population, but rather arise through an antigen-driven B cell response from an array of common naïve B cells [24].

It was originally believed that ABCs originate through an antigen-driven germinal center (GC) response, as they display somatically mutated heavy and light chains and isotype switching [24]. However, somatic hypermutation and class-switch recombination is known to take place outside of the GC [25–27]. Recent evidence indicates that ABCs can arise outside of the GC. Fate mapping studies have shown that a portion of ABCs are not GC-derived [28]. B cells outside of the GC express elevated phosphorylated signal transducer and activator of transcription 1 (pSTAT1), upstream of T-bet expression, in B cells [17], indicating that T-bet is likely up-regulated in B cells prior to GC entry [11]. It is possible, and has been previously suggested, that ABCs might undergo different differentiation processes and could arise from both within and outside of the GC [3]. Moving forward, fate mapping and imaging techniques will be important for determining the origination and precise anatomical localization of ABCs.

### Functions of ABCs during primary viral responses

ABCs display various functional capacities during viral infections, including antibody and cytokine production and interaction with T cells (Table 2). The production of antiviral antibodies is likely a major way in which ABCs contribute to the control of viral infections. T-bet expression in mouse B cells is required for IgG2a/c class switching and ABCs are enriched for IgG2a/c [29,30]. IgG2a/c (IgG1 in humans) is associated with a Th1 response and is the major antiviral isotype [31,32]. Knocking out ABCs in mice leads to a significant decrease in IgG2a/c titers [11–13], and transfer of virus-specific antibodies into mice deficient in ABCs is able to partially restore control of chronic LCMV [12].

**Table 2:** ABCs display multiple functional capacities during viral infections.

Antibodies	Express Rhinovirus-specific IgG in people [6]
	Class switch to IgG in chronic HCV in people [5]
	Express increased IgG1 and IgG3 in healthy individuals [7]
	Produce influenza, LCMV, or γHV68-specific IgG2a/c antibodies in mice [10–13]
Cytokines produced	Produce IFNγ, TNF, and IL-6 in uninfected mice [33–35]
	Express more IL-6 and TNF than follicular B cells in uninfected mice [35]
	Increased expression of IFNγ and TNF during γHV68 infection compared to naïve mice [36,37]
Interaction with T cells	Located at the T cell/B cell border in naïve mice [38]
	Increased effectiveness of antigen presentation compared to follicular B cells during influenza infection [11]

ABC, age-associated B cell; HCV, hepatitis C virus; IFNγ, interferon gamma; IgG, immunoglobulin G; IL-6, interleukin 6; LCMV, lymphocytic choriomeningitis virus; TNF, tumor necrosis factor; γHV68, gammaherpesvirus-68

B cells also contribute to antiviral immunity through mechanisms other than antibody production, including cytokine secretion and presentation of antigen to T cells [39]. ABCs secrete an array of cytokines with substantial production of IFNγ, tumor necrosis factor (TNF), and interleukin 6 (IL-6) [33–35]. ABCs express more IL-6 and TNF than follicular B cells, and ABC production of IL-6 and TNF is increased in old mice (18 to 22 months) compared to young mice (3 to 4 months) [35].

ABCs can interact with and stimulate T cells [11,38], though whether ABCs are acting as primary antigen-presenting cells or are reactivating T cells in a paracrine manner is less clear. Most evidence of ABC interaction with T cells comes from studies in the contexts of inflammatory and autoimmune diseases, rather than viral infection. In individuals with Crohn’s disease, T-bet^+^ B cell numbers correlate with IFNγ^+^ T cell numbers in the gut [40]. Further, culturing ABC-like cells from individuals with Crohn’s disease with autologous CD4^+^ T cells results in the production of IFNγ and IL-12 by the T cells [40]. This result highlights a role for ABCs in stimulating inflammatory T cells and suggests this as a possible way in which ABCs could contribute to disease. Similarly, systemic lupus erythematosus (SLE) mice without ABCs display defects in T cells, with fewer activated/memory CD4^+^ T cells and less IFNγ^+^CD8^+^ T cells compared to mice with ABCs [41]. Coculturing ABC-like cells from mice with autoimmune experimental hepatitis with CD4^+^ T cells resulted in impaired T cell proliferation and decreased IFNγ production [42]. In contrast, our group and others have reported no differences in IFNγ production by T cells between mice with and without ABCs during chronic LCMV and latent γHV68 infections [12,37]. During influenza infection CD11c^+^ B cells localize to the T cell–B cell boundary in the spleen and more efficiently present antigen than follicular B cells [11]. Although ABCs are capable of various antiviral functions, including antibody and cytokine secretion and antigen presentation to T cells, the relative contributions of these responses during and after viral infection are unclear and deserve further study.

### ABCs persist and play a role in memory and recall responses

In addition to responding to acute viral infection, ABCs persist following clearance of viral infections and appear to play a role in memory and recall responses. ABCs are a relatively stable population overtime; lineage tracing experiments show that T-bet^+^ B cells undergo minimal interconversion with T-bet^−^ cells [10]. Pathogen-specific ABCs persist long term in the spleen (Fig 1) through at least 100 days postinfluenza infection [10]. ABCs persist at elevated frequency in the spleen during γHV68 latency, at least 150 days postinfection [37]. ABCs play an important role in the control and clearance of chronic virus infection. Mice lacking ABCs are unable to clear chronic LCMV from the serum and display elevated viral load in the kidney compared to mice with ABCs [12].

Current evidence indicates that ABCs are important for recall responses. Without ABCs, mice display markedly decreased flu-specific IgG2c titers 40 days postinfection [10]. Upon rechallenge with a previously exposed antigen, ABCs tend to differentiate into antibody-secreting cells [10,16]. Clonal analysis demonstrates that, following influenza vaccination in people, ABCs are related to plasma cells [14]. ABC-derived antibody-secreting cells have been shown to be required for an effective recall response to influenza in mice [16]. The fate of these antibody-secreting cells following clearance of the challenge infection, and if/how the memory-like ABC population is replenished, is not currently known.

## Discussion

There are many interesting questions about ABC biology and their role in antiviral responses and disease still to be answered. Here, we discuss challenges and possibilities for future investigation.

### Functional contributions of ABC markers

The markers used to define the ABC subset, T-bet and CD11c among them, are currently more defining phenotypic markers rather than indicators of functional capacity. T-bet is a hallmark ABC marker, and T-bet expression in B cells has been shown to reliably differentiate between subsets that display unique anatomical localization patterns and antigen specificity profiles [10]. However, T-bet might not be required for the generation or functioning of ABCs. Du and colleagues showed that B cells deficient in T-bet can up-regulate ABC markers CD11c and CD11b in response to appropriate TLR and cytokine signals ex vivo and that these CD11c^+^CD11b^+^T-bet^−^ ABCs secrete the same quantity of IgM and IgG antibodies as ABCs with T-bet [43]. Furthermore, this group reports that mice with B cells deficient in T-bet can generate CD11c^+^CD11b^+^ ABC-like B cells in a model of lupus [43]. Alternatively, other groups, including our own, have shown a marked loss in CD11c-expressing ABCs in mice with T-bet-deficient B cells [41,44]. This indicates that CD11c expression is not necessarily a downstream effect of T-bet in all circumstances, but rather CD11c expression increases as a result of appropriate stimuli. CD11c is an integrin that, together with CD18, forms the CR4 complex that plays important roles in cellular adherence, migration, and phagocytosis [45]. Ultimately, the requirement of CD11c for ABC migration and functional capacities remains unclear. Moving forward, there should be further comparison of different ABC-like populations and examination of the functional roles of hallmark ABC markers.

### Phenotypic and functional differences of ABCs between contexts

In addition to viral infection, ABCs also expand in the contexts of female aging, autoimmunity, and malaria [1,2,46]. One major topic that requires further research is heterogeneity of the ABC population between contexts. Recently, substantial overlap in the transcriptional profiles of ABCs between individuals with malaria, HIV, and SLE was shown [47], though some differences were identified. Additional side-by-side comparisons of the ABC population between the contexts of aging, autoimmunity, and infection would be valuable. Beyond transcriptional profiles, analysis of functional capacities of ABCs between contexts is required. For instance, Rubstova and colleagues elegantly showed that ABCs are required for GC formation in the context of SLE, but not a model antigen [41]. Functional studies such as these will further elucidate contextual differences in ABC capacity.

### ABCs in localized and latent infections

The viral infections in which ABCs have thus far been implicated require systemic immune responses for clearance. However, whether ABCs play a role in infections cleared by more localized responses (for instance, enteric infections) is currently unclear.

There is robust evidence that ABCs play important roles in clearing acute and chronic infections, though their role in latent infections is less well defined. ABCs expand during latent viral infections including HIV, γHV68, and MCMV [7,8,13]. However, the role(s) they might play in the latency phase of infection is not well understood. Gammaherpesvirus infections such as γHV68 and Epstein–Barr virus infect B cells, but whether ABCs are susceptible to direct infection with these latent viruses is not yet known. Accordingly, it is also unknown whether ABCs serve as a viral reservoir to maintain latency. During latency, viruses deploy a distinct transcription profile in which very few genes are expressed, and viral replication is substantially reduced. A continuous immune response persists during latency that is distinct from that of acute infection and keeps the latent virus in check while modulating immune responses to other antigens [48]. Latency is maintained through a combination of humoral and cellular immunological factors and whether ABCs play a role in this détente between the host immune system and latent infection is unclear.

### Sex differences in ABCs

It is well established that there exist sex differences in responses to viral infection [49]. ABCs display a sex bias, in which their numbers and proportions are increased in females more so than males, during both aging [1] and autoimmunity [36,50]. Currently, it is not clear if ABCs display a sex bias during viral infection and, if so, what the impact might be on control of infection and resulting immunopathology between females and males. It has been hypothesized that ABCs display a female sex bias due to their requirement for X chromosome–linked TLR7 signaling for formation [51]. TLR7, stimulated primarily by viral single-stranded RNA, is encoded by the X chromosome and is susceptible to incomplete inactivation in females, leading to higher TLR7 expression in females [52]. In support of the theory that the ABC sex bias is due to increased TLR7 expression in females, *Tlr7* duplication in male mice resulted in a significant increase in ABC frequency [50]. In addition to a sex difference in the frequency of ABCs, it was recently shown that ABCs in male versus female mice display altered functional capacities in the context of SLE [50]. In particular, ABCs from female mice secreted significantly more self-specific IgG2a/c and were enriched for interferon response pathways compared to those from males [50]. It remains to be seen if these ABC functional differences between males and females are present during viral infection and if this could contribute to the well-reported sex differences to viral infections [49].

### ABCs as mediators between viral infection, autoimmunity, and aging

In addition to the independent expansion of ABCs across contexts, there is evidence that ABCs may function as mediators between viral infection and autoimmunity. HCV-induced ABC-like cells produce rheumatoid factor-type autoantibodies [53]. Additionally, we have recently demonstrated that T-bet^+^ B cells are required for ɣHV68 exacerbation of arthritis and EAE [36,44]. It is well established that the ABC population expands with age [1,34,35], but how this increased abundance impacts viral infection and autoimmunity remains understudied. ABCs in aged mice secrete more autoreactive antibodies [35] and proinflammatory markers, including TNF and IL-6, than ABCs in young mice [35]. How these changes to the ABC population with age impact their contribution to autoimmunity or control of viral infections deserves further investigation.

## Conclusions

There is ample evidence that ABCs play a role in an array of viral infections during both the acute and chronic stages (Box 2). ABCs are a relatively rare persistent effector subset that display various unique characteristics, from activation requirements and localization patterns to transcriptional profile and functional characteristics that differ from other B cell subsets. The ABC population transiently increases in circulation during acute viral infection and persists indefinitely in the spleen following infection resolution. Various functional capacities are exerted by ABCs, in particular the secretion of antibodies and cytokines and activation of T cells. The contributions of ABCs to immune homeostasis and disease remain areas of intense investigation and their continued investigation in in vivo models and human samples will be critical for further elucidating their immunobiology and precise mechanisms of contribution.

**Box 2. Key papers**

1. Rubtsova K, Rubtsov AV, van Dyk LF, Kappler JW, Marrack P. T-box transcription factor T-bet, a key player in a unique type of B-cell activation essential for effective viral clearance. Proc Natl Acad Sci. 2013;**110**:E3216–E3224.
2. Barnett BE, Staupe RP, Odorizzi PM, Palko O, Tomov VT, Mahan AE, et al. B cell intrinsic T-bet expression is required to control chronic viral infection. J Immunol Baltim Md 1950. 2016;**197**:1017–1022.
3. Knox JJ, Buggert M, Kardava L, Seaton KE, Eller MA, Canaday DH, et al. T-bet^+^ B cells are induced by human viral infections and dominate the HIV gp140 response. JCI Insight. 2017;**2**. doi: 10.1172/jci.insight.92943
4. Lau D, Lan LY-L, Andrews SF, Henry C, Rojas KT, Neu KE, et al. Low CD21 expression defines a population of recent germinal center graduates primed for plasma cell differentiation. Sci Immunol. 2017;**2.** doi: 10.1126/sciimmunol.aai8153
5. Johnson JL, Rosenthal RL, Knox JJ, Myles A, Naradikian MS, Madej J, et al. The Transcription Factor T-bet Resolves Memory B Cell Subsets with Distinct Tissue Distributions and Antibody Specificities in Mice and Humans. Immunity. 2020. doi: 10.1016/j.immuni.2020.03.020
